# An Energy-Based Concept for Yielding of Multidirectional FRP Composite Structures Using a Mesoscale Lamina Damage Model

**DOI:** 10.3390/polym12010157

**Published:** 2020-01-07

**Authors:** Seyed Saeid Rahimian Koloor, Atefeh Karimzadeh, Noorfaizal Yidris, Michal Petrů, Majid Reza Ayatollahi, Mohd Nasir Tamin

**Affiliations:** 1 Institute for Nanomaterials, Advanced Technologies and Innovation, Technical University of Liberec, Studentska 2, 461 17 Liberec, Czech Republic; 2 Department of Aerospace Engineering, Faculty of Engineering, Universiti Putra Malaysia, Serdang 43400, Malaysia; 3 School of Mechanical Engineering, Faculty of Engineering, Universiti Teknologi Malaysia, Johor Bahru 81310, Malaysia; a.karimzadeh.66@gmail.com; 4 Fatigue and Fracture Research Laboratory, Center of Excellence in Experimental Solid Mechanics and Dynamics, School of Mechanical Engineering, Iran University of Science & Technology, Tehran 16846, Iran; m.ayat@iust.ac.ir

**Keywords:** composite structures, multidirectional FRP composite laminates, composite yielding, damage dissipation energy, continuum damage model, finite element simulation

## Abstract

Composite structures are made of multidirectional (MD) fiber-reinforced polymer (FRP) composite laminates, which fail due to multiple damages in matrix, interface, and fiber constituents at different scales. The yield point of a unidirectional FRP composite is assumed as the lamina strength limit representing the damage initiation phenomena, while yielding of MD composites in structural applications are not quantified due to the complexity of the sequence of damage evolutions in different laminas dependent on their angle and specification. This paper proposes a new method to identify the yield point of MD composite structures based on the evolution of the damage dissipation energy (DDE). Such a characteristic evolution curve is computed using a validated finite element model with a mesoscale damage-based constitutive model that accounts for different matrix and fiber failure modes in angle lamina. The yield point of composite structures is identified to correspond to a 5% increase in the initial slope of the DDE evolution curve. The yield points of three antisymmetric MD FRP composite structures under flexural loading conditions are established based on Hashin unidirectional (UD) criteria and the energy-based criterion. It is shown that the new energy concept provides a significantly larger safe limit of yield for MD composite structures compared to UD criteria, in which the accumulation of energy dissipated due to all damage modes is less than 5% of the fracture energy required for the structural rupture.

## 1. Introduction

The ever-increasing use of advanced polymer composites such as fiber-reinforced polymer (FRP) composites, nanocomposites, etc., as high strength-to-weight ratio and high-stiffness structural materials in advanced industrial applications [1,2,3], presents a unique design challenge with highly anisotropic material responses of the composites [4,5,6,7,8,9]. The FRP composites are essentially brittle with no plastic deformation, thus adequately described using a bilinear stress–strain curves with elastic and softening responses. The softening response represents the permanent deformation of the material, described through the continuous degradation of stiffness and strength to complete fracture [10,11,12,13]. The general loading on composite structures, including axial, flexural, and torsional loads, generates a complex stress state in the material. Consequently, a mechanics-equivalent stress quantity is determined in comparison with the corresponding property at the yield of the material [14,15,16,17,18,19,20]. The Tsai–Wu theory (1970), as one of the first failure criteria, was established based on Hill’s theory of the macroscopic yielding of anisotropic metals, to predict the yielding of composite materials [21,22]. Thereafter, many composite failure criteria, including Hashin (1980), Chang–Lessard (1991), Puck (1998), Cuntze (2004), Azizi (2012), and Daniel (2015), were formulated in the form of stress-based relations to predict the yielding/failure of composites in the mixed-mode condition [22,23,24,25,26,27,28,29]. The Christensen macroscopic yield criterion for fibrous composites consists of two quadratic stress-based equations for failure prediction of the matrix and fibers [30]. Lissenden, through an empirical approach, established the initial and subsequent yield surfaces of metal matrix composite (MMC) laminates, and characterized the hardening behavior [31]. Azizi et al. used strain gradient plasticity of the fiber/matrix unit cell of a continuous fiber composite laminate to develop the anisotropic pressure-dependent yield function for MMCs in the macroscale [28]. A lamina strain rate yield model in the form of the master failure envelope under multi-axial sates of stress was established [29,32]. Some of these failure criteria are employed in finite element (FE) codes, and they are used to predict the damage and failure processes in composite materials [33,34].

In the continuum damage mechanics approach, the gradual elastic softening deformation of the composite lamina, which represents the multiple damage processes, could be used to quantify the local permanent deformation and yielding nature in the complex stress state of the composite lamina [10,35]. In this approach, the fibrous composite yield models are treated as a criterion of damage initiation of composite lamina in the mesoscale [10,36]. The subsequent damage propagation or softening process of the composite is described using energy-based models [37,38]. Maimi et al. proposed a damage model for intralaminar failure and collapse of the composite structure. The model was updated to consider the effect of fiber rotation during the loading, which resulted in a stiffer composite structure against loading [38,39]. Zhuang et al. developed a numerical model for bearing damage of composite laminates in the mesoscale to predict the size and shape of the damage zones [37]. Fakoor et al. performed a numerical and experimental study on the progressive damage of a composite laminate, considering both linear and various exponential softening laws, to address the first and last ply failures (FPF and LPF) [40]. Su et al. employed a progressive damage model to describe the compression response of the open-hole composite laminate, considering both in-plane and out-of-plane deformations [41]. A gradual stiffness degradation model was proposed by Yang et al. for the estimation of damage evolution in composite laminates under three-point bending load [42]. Cherniaev et al. used a three-damage-based constitutive model to simulate the crushing response of composite tube, considering bilinear softening for fiber damage, matrix damage with linear softening, and hardening-softening due to shear damage [35]. R. Koloor et al. developed a mesoscale damage evolution model for angle lamina incorporating Hashin failure criteria [24] and an energy-based damage model [10] for the prediction of the damage and post-damage initiations, as well as the subsequent softening process [10,43,44,45].

In industrial scale, composite structures are made of MD composite laminate, in which their design basis uses the reviewed criteria that were introduced as the yield limit, damage, and failure of unidirectional (UD) composite lamina level, rather than the structural level. In the UD FRP composites, the calculated ultimate strength based on measured data from a standard test [10] is often treated as the yield strength of the composite material. The strength is determined based on tensile and compressive stresses, as well as shear stress in the fiber and matrix directions [10,46]. In MD FRP composite laminates, matrix yielding/cracking and interface delamination are observed as the early damage mechanisms due to very low stiffness/strength properties of the matrix and interface (10%–20%) compared to the fiber properties level [10,47,48,49,50]. In addition, the variation of through-thickness lamina orientations in an MD composite facilitates matrix failure and multi-delamination events at the early stage of the loading [10,43,45,51]. In some cases, the MD FRP composite structure was able to sustain up to 10-fold higher loading above the level corresponding to the onset of matrix and interface damages [10,43,45]. Accordingly, considering the UD lamina level yield criteria to estimate the failure of an MD composite structure normally results in the prediction of structural failure at 5%–10% maximum load capacity of the structure [2,10,43,44,52], which prevents the optimum design of light composite structures. Therefore, material and loading-related parameters should be developed to provide a consistent identification of the yield point for the MD FRP composite structures [2,5,10,43,44,45].

In this study, an energy concept is developed based on the energy dissipated during the inelastic deformation process of lamina using a mesoscale damage model, to estimate the yield of MD FRP composite structures. The critical level of the accumulated damage dissipation energy (DDE) in FRP composite laminate is proposed as the parameter that indicates the yield of the material. The continuum damage mechanics that account for damage initiation and propagation in the material point are used to quantify the material softening process and compute the rate of DDE growth, to establish the critical DDE level. The characteristic evolution of the DDE is established through FE simulations of actual tests using a validated FE model. The yield point is inferred from the DDE curve, when a sudden increasing rate is observed. The approach is illustrated for different types of antisymmetric MD FRP composite structures with the objective of determining the yield strength. The experiments were implemented on carbon and glass fiber-reinforced polymer (CFRP and GFRP) composite structures, such that only lamina damage could occur with negligible interface delamination; therefore, interlaminar damage was not considered [10,43]. A new approach with FE model-based configurations called single- and multi-layer models [10] was used to simulate the FRP composites manufactured using different methods, to model the mesoscale inter- and intralaminar constructions of the composites. The simulation results were validated with the structural response of the composites in the experiments. The method is recommended for determining the yield limit of any type of MD composite structure under different types of load.

## 2. Damage Model of FRP Composite Lamina

The response of the FRP composite laminates to applied load such that yielding of the material is achieved is predicted in this study, based on the damage mechanics approach. The damage model of the FRP composite lamina is described below.

**Figure 1:**
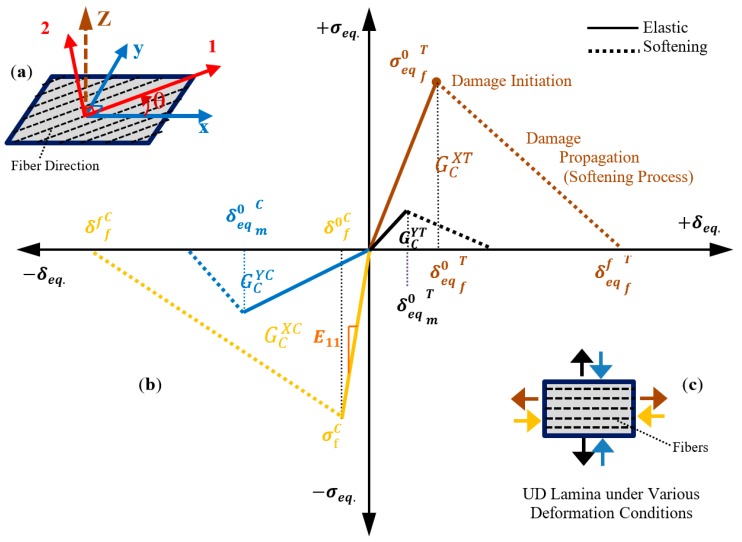
(**a**) Local (1–2) and global (*x*–*y*) axes of an angle lamina; (**b**) bilinear stress–strain behavior of fiber-reinforced polymer (FRP) lamina in orthogonal axes for various failure modes. (**c**) Each colored curve corresponds to the loading as shown by the same colored arrows in the inset figure.

The uniaxial behavior of UD FRP composite lamina in orthogonal axes (1–2 axis, Figure 1a) for elastic-damage behavior under tension and compression is shown in Figure 1b. The four bilinear elastic softening curves represent the equivalent stress–displacement behavior of composite lamina in different failure modes of matrix cracking and crushing, and fiber breakage and buckling (following the load arrows of UD lamina, Figure 1b). In an angle lamina under global loading (*x–y* axis, Figure 1a), the global deformations are mapped into local deformation and used to compute the effective stress parameters. The elastic behavior of the lamina is computed following the classical theory of lamina [53,54]. The stress–displacement relationship of each damage mode is defined in the sections below.

### 2.1. Damage Initiation

The initiation of damage in the lamina for the different failure modes is estimated using Hashin’s quadratic stress-based failure model [24]. The model is expressed as a quadratic function of the ratio of the effective stress to strength terms to calculate the values of damage variables for the respective failure mode.

Matrix cracking and crushing:

(1) $${\left(\frac{{\hat{\sigma }}_{22}}{Y^{T}}\right)}^{2}+{\left(\frac{{\hat{\tau }}_{12}}{S^{L}}\right)}^{2}=d_{m}^{t};\;for\;{\hat{\sigma }}_{22}\geq 0\;\left(Tension\right).$$

(2) $${\left(\frac{{\hat{\sigma }}_{22}}{2S^{T}}\right)}^{2}+\left[{\left(\frac{Y^{C}}{2S^{T}}\right)}^{2}-1\right]\left(\frac{{\hat{\sigma }}_{22}}{Y^{C}}\right)+{\left(\frac{{\hat{\tau }}_{12}}{S^{L}}\right)}^{2}=d_{m}^{c};\;for\;{\hat{\sigma }}_{22}<0\;\left(Compression\right).$$

Fiber fracture and buckling/kinking:

(3) $${\left(\frac{{\hat{\sigma }}_{11}}{X^{T}}\right)}^{2}+{\left(\frac{{\hat{\tau }}_{12}}{S^{L}}\right)}^{2}=d_{f}^{t};\;for\;{\hat{\sigma }}_{11}\geq 0\;\left(Tension\right).$$

(4) $${\left(\frac{{\hat{\sigma }}_{11}}{X^{C}}\right)}^{2}=d_{f}^{c};\;for\;{\hat{\sigma }}_{11}<0\;\left(Compression\right)$$

In the equations above, $\left[\hat{\sigma }\right]$ represents the effective stresses in the lamina, and *X^T^, Y^T^, X^C^, Y^C^, S^L^,* and *S^T^* are the strength properties. In Equations (1)–(4), $d_{f}^{t},$ $d_{f}^{c}$ and $d_{m}^{t},$ $d_{m}^{c}$ are the internal damage variables in the fiber and matrix phases of the lamina, under tension or compression loadings. Since no plastic deformation is observed in the FRP composite [2,10,55], the permanent deformation of the lamina is considered in the damage evolution processes.

*Post-damage initiation:*

Once the onset of damage is predicted in one of the modes, the properties of the material reduce in the other directions/modes and result in early damage. Such effects could be captured by updating the elastic stress tensor (effective stresses in Equations (1)–(4) through internal damage variables.

(5) $${\hat{\sigma }}_{ij}=\{\begin{array}{c} \sigma_{ij}\;Prior\;to\;any\;damage\;initiation\\ D\;\sigma_{ij}\;If\;any\;of\;the\;four\;damages\;has\;initiated \end{array}$$

where σ is the stress computed using classical lamina theory, $\left[\hat{\sigma }\right]$ is the effective stress in Equations (1)–(4), and the damage operator, D, is used to consider the effect of early damage initiations. The hypothesis of strain equivalence is used to derive the damage operator as follows [54,56]:

(6) $$D=\left[\begin{array}{ccc} 1/\left(1-d_{f}\right) & 0 & 0\\ 0 & 1/\left(1-d_{m}\right) & 0\\ 0 & 0 & 1/\left(1-d_{s}\right) \end{array}\right]$$

where $d_{f},{\;d}_{m},$ and $d_{s}$ are the fiber, matrix, and shear internal damage variables corresponding to the lamina damage modes in Equations (1)–(4).

### 2.2. Damage Propagation

**Figure 2:**
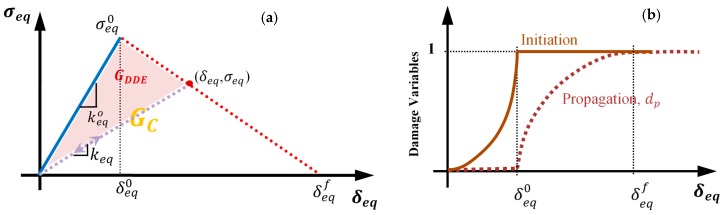
(**a**) Damage dissipation energy in the stress–displacement curve; (**b**) evolution of the damage initiation (solid brown line) and propagation (dotted brown line) variables at the material point.

The evolution of damage to failure at a local material point is obtained through the softening process using energy-based criterion [10,43,45]. In this process, the damage dissipation energy, $G_{DDE}$ (Figure 2a), is employed to determine the constitutive model of the material in each failure mode, which is expressed as the stress–displacement relation. The fracture energy, $G_{C},$ is the energy that, if dissipated fully, causes the material to fail $(G_{C}^{XT},$ $G_{C}^{XC},$ $G_{C}^{YT},$ and $G_{C}^{YC}$ are the fracture energies in different failure modes; Figure 1b). The value of dissipated energy due to damage is obtained using

(7) $$G_{DDE}=\frac{1}{2}d_{p}\;k_{eq}^{0}\;\delta_{eq}^{o}\;\delta_{eq}$$

with the corresponding damage evolution variable, *d_p_*, defined as

(8) $$d_{p}=\frac{\delta_{eq}^{f}\left(\delta_{eq}-\delta_{eq}^{0}\right)}{\delta_{eq}\left(\delta_{eq}^{f}-\delta_{eq}^{0}\right)}\;\delta_{eq}\geq \delta_{eq}^{0}$$

where $k_{eq}^{0}$ is the equivalent elastic stiffness, $\delta_{eq}^{0}$ is the equivalent displacement at the onset of damage in the respective mode $\left(d_{p}=0\right),$ and $\delta_{eq}^{f}$ is the equivalent displacement at the separation of the material point $\left(d_{p}=1\right).$ In each failure mode, the critical value of equivalent dissipation energy, $G_{C},$ is considered as the fracture energy of the lamina. The evolutions of the damage initiation variable $(d_{i}$ in Equations (1)–(4)) and damage propagation variable $\left(d_{p}\right)$ are shown in Figure 2b.

The relation between the equivalent stress–displacement for each failure mode, after onset of damage (dotted lines in Figure 1b,c), is expressed by the equations below [10,43,44,45].

Matrix tension $\left({\hat{\sigma }}_{22}\geq 0\right)$:

(9) $$\sigma_{eq.}=\left(\frac{{\left(\langle \sigma_{22}^{o}\rangle \langle \varepsilon_{22}^{o}\rangle +\tau_{12}^{o}\varepsilon_{12}^{o}\right)}^{2}}{\left(\left(L^{c}\left(\langle \sigma_{22}^{o}\rangle \langle \varepsilon_{22}^{o}\rangle +\tau_{12}^{o}\varepsilon_{12}^{o}\right)\right)-2G_{C}^{YT}\right)\times \left(\langle \varepsilon_{22}^{o}\rangle {}^{2}+\varepsilon_{12}^{o}{}^{2}\right)}\right)\times \left(\delta_{eq.}-\frac{2G_{C}^{YT}\;\sqrt{\langle \varepsilon_{22}^{o}\rangle {}^{2}+\varepsilon_{12}^{o}{}^{2}}}{\langle \sigma_{22}^{o}\rangle \langle \varepsilon_{22}^{o}\rangle +\tau_{12}^{o}\varepsilon_{12}^{o}}\right)$$

Matrix compression $\left({\hat{\sigma }}_{22}<0\right)$:

(10) $$\sigma_{eq.}=\left(\frac{{\left(\langle -\sigma_{22}^{o}\rangle \langle -\varepsilon_{22}^{o}\rangle +\tau_{12}^{o}\varepsilon_{12}^{o}\right)}^{2}}{\left(L^{c}\left(\langle -\sigma_{22}^{o}\rangle \langle -\varepsilon_{22}^{o}\rangle +\tau_{12}^{o}\varepsilon_{12}^{o}\right)-2G_{C}^{YC}\;\right)\left(\langle -\varepsilon_{22}^{o}\rangle {}^{2}+\varepsilon_{12}^{o}{}^{2}\right)}\right)\times \left(\delta_{eq.}-\frac{2G_{C}^{YC}\;\sqrt{\langle -\varepsilon_{22}^{o}\rangle {}^{2}+\varepsilon_{12}^{o}{}^{2}}\;}{\langle -\sigma_{22}^{o}\rangle \langle -\varepsilon_{22}^{o}\rangle +\tau_{12}^{o}\varepsilon_{12}^{o}}\right)$$

Fiber tension $\left({\hat{\sigma }}_{11}\geq 0\right)$:

(11) $$\sigma_{eq.}=\left(\frac{{\left(\langle \sigma_{11}^{o}\rangle \langle \varepsilon_{11}^{o}\rangle +\tau_{12}^{o}\varepsilon_{12}^{o}\right)}^{2}}{\left(\left(L^{c}\left(\langle \sigma_{11}^{o}\rangle \langle \varepsilon_{11}^{o}\rangle +\tau_{12}^{o}\varepsilon_{12}^{o}\right)\right)-2G_{C}^{XT}\right)\times \left(\langle \varepsilon_{11}^{o}\rangle {}^{2}+\varepsilon_{12}^{o}{}^{2}\right)}\right)\times \left(\delta_{eq.}-\frac{2G_{C}^{XT}\;\sqrt{\langle \varepsilon_{11}^{o}\rangle {}^{2}+\varepsilon_{12}^{o}{}^{2}}}{\langle \sigma_{11}^{o}\rangle \langle \varepsilon_{11}^{o}\rangle +\tau_{12}^{o}\varepsilon_{12}^{o}}\right)$$

Fiber compression $\left({\hat{\sigma }}_{11}<0\right)$:

(12) $$\sigma_{eq.}=\left(\frac{\langle -\sigma_{11}^{o}\rangle {}^{2}}{\left(L^{c}\langle -\varepsilon_{11}^{o}\rangle \langle -\sigma_{11}^{o}\rangle -2G_{C}^{XC}\right)}\right)\times \left(\delta_{eq.}-\frac{2G_{C}^{XC}\;}{\langle -\sigma_{11}^{o}\rangle }\right)$$

In these equations, $L^{c}$ is the element characteristic length with magnitude depending on the geometry and the element formulation. For the first-order element, $L^{c}$ is considered as the length of a line across the element. The terms $G_{C}^{XT},$ $G_{C}^{XC},$ $G_{C}^{YT},$ and $G_{C}^{YC}$ are the fiber and matrix fracture energy parameters of the lamina under tension and compression loadings. In Equations (9)–(12), $\sigma_{ij}^{o},$ $\tau_{ij}^{o},$ and $\varepsilon_{ij}^{o}$ indicate the effective stresses at the onset of damage [10,43].

### 2.3. Damage Dissipation Energy

The energy stored in the FRP composite laminate through elastic-damage deformation, commonly called the internal energy, can be employed to describe the progressive damage process of the composite structure [10,43,45,57]. The internal energy, $E_{U},$ can be written for non-viscous composites as

(13) $$E_{U}=\int_{0}^{t}\left(\int_{V}^{.}\sigma^{c}:\dot{\varepsilon }\;dV\right)dT$$

where $\sigma^{c}$ is the stress derived from the constitutive equation of a lamina. The strain rate term is decomposed as

(14) $$\dot{\varepsilon }={\dot{\varepsilon }}^{el}+{\dot{\varepsilon }}^{pl}+{\dot{\varepsilon }}^{cr}$$

where ${\dot{\varepsilon }}^{el},\;{\dot{\varepsilon }}^{pl},\;and\;{\dot{\varepsilon }}^{cr}$ are the time rates of elastic, plastic, and creep strains, respectively. Since FRP lamina behaves in the form of elastic-brittle material, thus ${\dot{\varepsilon }}^{pl}={\dot{\varepsilon }}^{cr}=0,$ and Equation (13) can be simplified to

(15) $$E_{S}=\int_{0}^{t}\left(\int_{V}^{.}\sigma^{c}:{\dot{\varepsilon }}^{el}dV\right)dT$$

where $E_{S}$ is the elastic strain energy. The elastic strain is not recoverable when damage initiates in a material point. Hence, $\sigma^{c}$ can be expressed in the following form:

(16) $$\sigma^{c}=\left(1-d\right)\sigma^{u},\;d\in \left[0,1\right]$$

where $\sigma^{u}$ is un-damaged stress, and d is the continuum damage parameter which varies from “zero” for the undamaged state to “one” for the fully damaged state of the material point in the composite lamina. Therefore, substituting $\sigma^{c}$ into Equation (15) gives elastic strain energy as

(17) $$E_{S}=\int_{0}^{t}\left(\int_{V}^{.}\left(1-d\right)\sigma^{u}:{\dot{\varepsilon }}^{el}dV\right)dT$$

It is assumed that the damage parameter remains fixed at time *t* until unloading. Thus, the recoverable strain energy and the dissipated energy during damage can be expressed as follows:

(18) $$E_{E}=\int_{0}^{t}\left(\int_{V}^{.}\left(1-d_{t}\right)\sigma^{u}:{\dot{\varepsilon }}^{el}dV\right)dT=\int_{0}^{t}\left(\int_{V}^{.}\left(\frac{1-d_{t}}{1-d}\right)\sigma^{c}:{\dot{\varepsilon }}^{el}dV\right)dT$$

(19) $$E_{D}=\int_{0}^{t}\left(\int_{V}^{.}\left(d_{t}-d\right)\sigma^{u}:{\dot{\varepsilon }}^{el}dV\right)dT=\int_{0}^{t}\left(\int_{V}^{.}\;\left(\frac{d_{t}-d}{1-d}\right)\sigma^{c}:{\dot{\varepsilon }}^{el}dV\right)dT\;$$

Considering the undamaged elastic energy function $f^{u},$ interchanging the integrals in Equations (18) and (19) yields

(20) $$E_{E}=\int_{V}^{.}\left(\int_{0}^{t}\left(1-d_{t}\right){\dot{f}}^{u}dT\right)dV=\int_{V}^{.}\left(\left(1-d_{t}\right)f^{u}\right)\;dV$$

(21) $$E_{D}=\int_{V}^{.}\left(\int_{0}^{t}\;\left(d_{t}-d\right){\dot{f}}^{u}dT\right)dV=\int_{V}^{.}\left[\left(d_{t}-d\right)f^{u}{|}_{0}^{t}+\int_{0}^{t}\;\dot{d}f^{u}dT\right]dV\;$$

Since, at time t, $d=d_{t}$ and, at time zero, $f^{u}=0,$ the first term of the last expression in Equation (21) is zero. By defining the damage strain energy function $f^{c}$ as $\left(1-d_{t}\right)f^{u},$ Equations (20) and (21) can be written as follows:

(22) $$E_{E}=\int_{V}^{.}\left(\left(1-d_{t}\right)f^{u}\right)dV=\int_{V}^{.}f^{c}dV$$

(23) $$E_{D}=\int_{V}^{.}\left(\int_{0}^{t}\;\frac{\dot{d}}{1-d}\;f^{c}dT\right)dV=\int_{0}^{t}\;\int_{V}^{.}\frac{\dot{d}}{1-d}\;f^{c}dVdT\;$$

The parameter $f^{c}$ can be written for a linear elastic energy function as

(24) $$f^{c}=\frac{1}{2}\sigma^{u}:\varepsilon^{el}.\;$$

Substituting Equation (24) into Equations (22) and (23) gets

(25) $$E_{E}=\int_{V}^{.}\frac{1}{2}\sigma^{u}:\varepsilon^{el}dV$$

(26) $$\;E_{D}=\int_{0}^{t}\;\int_{V}^{.}\frac{\dot{d}}{2\left(1-d\right)}\;\sigma^{u}:\varepsilon^{el}dVdT\;$$

The present study employed these equations through FE simulations to establish the characteristic evolution of the DDE and to illustrate the proposed concept for determining the yield limit of MD FRP composite structures [43,45]. The yield point was identified corresponding to a 5% increase in the initial slope of the total DDE evolution curve of the composite structure under specific loading conditions.

## 3. Materials and Experimental Procedures

**Figure 3:**
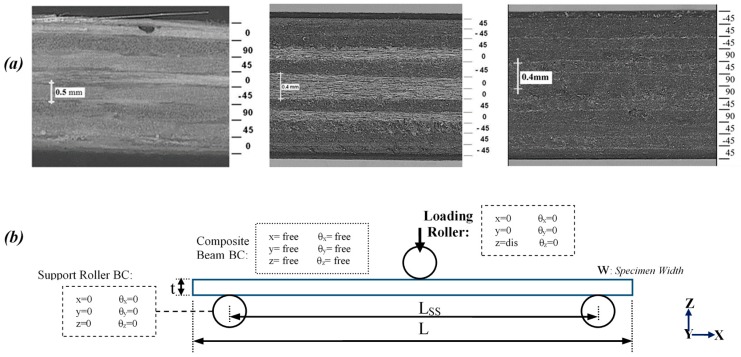
(**a**) Longitudinal cross-section of (left) glass fiber-reinforced polymer (GFRP) and (middle and right) carbon fiber-reinforced polymer (CFRP) composite specimens, (**b**) A schematic view of the composite beam on the test set-up.

Three different types of antisymmetric MD FRP composite laminate panels were manufactured and machined into beam specimen geometries for flexural tests. These tests were performed in accordance with the ASTM standard [58]. The antisymmetric MD composite lay-up, sample geometry, and load and boundary conditions were selected such that, under the flexural deformation, the various lamina failure modes under tension and compression were activated, while the interlaminar damage and delamination phenomenon were minimized. Subsequently, microscopic fractographic analysis was used to examine the lamina and interface damage events, which indicate dominant lamina damage with negligible interface delamination [10,43,45]. The first group of specimens was fabricated from a glass fiber-reinforced polymer (GFRP) composite panel with thermoplastic resin and eight layers of UD E-glass fiber mats. The GFRP composite was prepared using vacuum-assisted infusion molding (VAIM) process, which resulted in the formation of laminate with no interface between the laminas [10]. The next two groups of the composite samples were made of carbon fiber-reinforced polymer (CFRP) composite laminate. A panel of the CFRP composite was prepared by pre-impregnation of the UD CFRP lamina (M40J fibers and NCHM 6376 resin, Structil France) and cured in an autoclave, resulting in a composite laminate with interface laminas [10]. Details of the manufacturing process of the FRP panels were provided elsewhere [2,10,43]. Microscopic images of the longitudinal cross-section of the FRP composite specimens are shown in Figure 3a. A schematic view of the composite beam in the test set-up and boundary conditions is provided in Figure 3b, in which the lay-ups and dimensions of the beams, and the load configurations are described in Table 1. Several GFRP composite specimens and the first batch of the CFRP composite samples were tested under three-point bending (3PB) conditions, while the second batch of CFRP composite samples was tested under four-point bending (4PB), as mentioned in Table 1. A continuous flexural load was applied to the specimens until a significant amount of degradation in load–deflection response, which represented the occurrence of multiple damages, was observed. The results of the tests in terms of monotonic reaction force versus the deflection of the composite beams were recorded and utilized in the validation procedure of the FE models and simulation processes.

## 4. Finite Element Simulation

**Figure 4:**
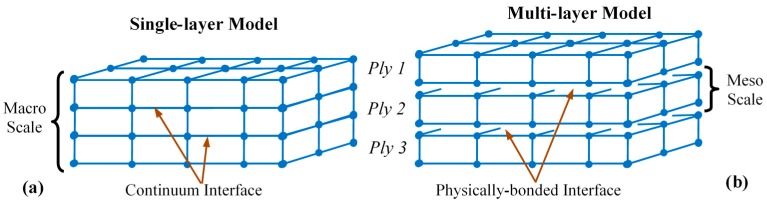
Configuration of (**a**) single-layer and (**b**) multi-layer finite element (FE) model-based constructions of FRP composite laminates for vacuum-assisted infusion molding (VAIM) and prepreg/autoclave manufacturing processes, respectively.

Since the manufacturing process of the FRP composite laminates dictates the resulting interface condition between the laminas, the FE models of these cases are different. The GFRP composite laminate, produced by the VAIM method with no apparent interface, was simulated with the single-layer model. The CFRP composite laminate, fabricated via lay-ups of prepreg laminas and autoclave curing with distinct interfaces, was modeled using the multi-layer model. The single-layer and multi-layer FE model constructions are illustrated in Figure 4, while details of these mesoscale FE models were discussed elsewhere [10,43,45].

**Figure 5:**
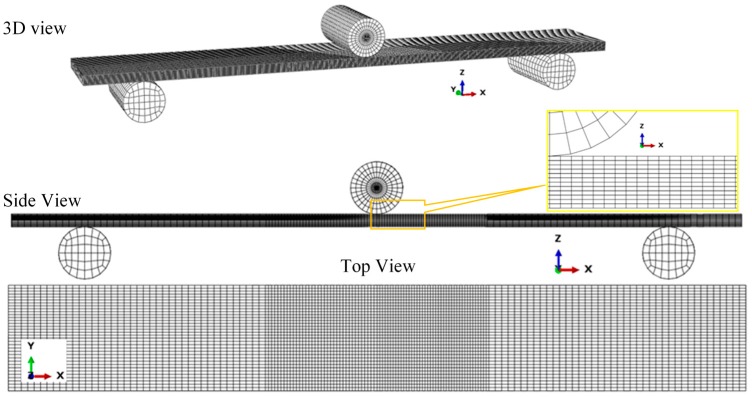
FE model of the CFRP composite specimen under the three-point bending (3PB) test set-up, showing the discretized geometry.

The three-dimensional (3D) FE model geometry of the CFRP composite laminate beam specimen is shown in Figure 5. The damage model (Section 2) was applied in each lamina using the standard model definition step in ABAQUS software [57]. Each lamina was discretized into a layer of eight-node continuum shell elements (SC8R) with reduced integration points for efficient computation [37]. The element mesh was refined with smaller-size elements, defined for the central region of the specimen when the maximum deflection and, thus, damage evolution was anticipated. The loading and support rollers were modeled as rigid bodies and discretized using rigid, four-node continuum elements (R3D4) [57].

Frictionless contact was assumed between all contacting bodies. In the multi-layer model (Figure 4b), the interfaces between adjacent laminas were modeled using a surface-to-surface tie with finite displacement interaction of the sharing node pair. This allows relative displacement between adjacent surfaces of the laminas [10,45]. A two-step mesh convergence process was performed to eliminate the effect of element size on the FE-calculated results for the elastic and damage calculations [43,45]. A finer element mesh size than that adequately identified in the elastic analysis is required for element size-independent damage calculations. The resulting element size, at mesh convergence, had an edge length of 0.2 mm. The boundary conditions of the model are illustrated in Figure 3b, while the loading was identical to that used during the test.

The elastic and strength properties of GFRP and CFRP composite laminates used in the FE simulations were obtained through standard tests (ASTM-D4762, [59]) [10,43,45], while the values of fracture energies were extracted from the properties of similar materials in the literature [60,61,62], as listed in Table 2. These properties were utilized rigorously in the FE simulation exercises of the various composite specimen geometries and loading cases, demonstrating comparable load–displacement results with measured responses [2,10,43,44,45,52].

## 5. Results and Discussion

The FE-calculated and measured flexural responses of the MD FRP composite beam specimens, expressed in terms of the load–deflection curves, were compared. A comparable response was indicative of the validity of the FE simulation procedures. Subsequently, the calculated deformation and damage responses of the composite laminates were interpreted for the respective failure mechanisms. The characteristic evolution of the DDE could then be established and inferred for the onset of yield of the composite structure.

### 5.1. Structural Response and Damage Evolution of GFRP Composite Beam under Three-Point Bending

**Figure 6:**
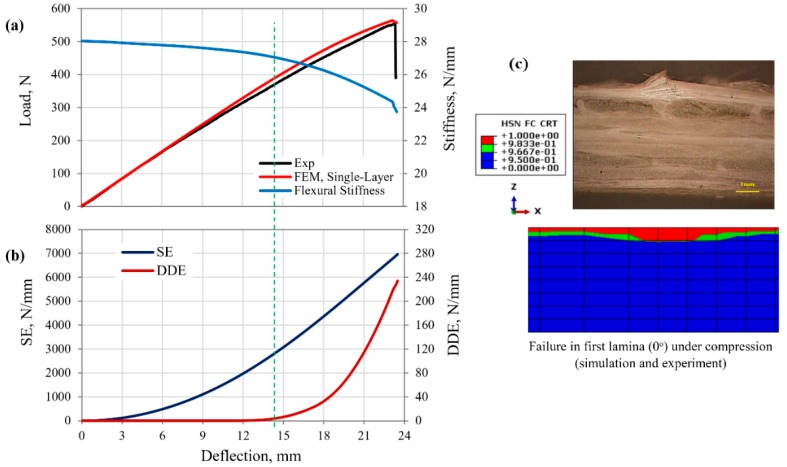
(**a**) Load–deflection response, structural stiffness curve, (**b**) strain and damage dissipation energies, and (**c**) final failure of GFRP composite beam under 3PB load.

The FE-calculated load–deflection response of the MD GFRP composite beam specimen (*Case 1*, Table 1) was compared with the measured curve, as shown in Figure 6a. The reasonably good prediction of the flexural response rendered the FE simulation valid. The GFRP composite beam showed an initial linear response up to the deflection of about 12 mm, suggesting structural elastic behavior; however, the flexural stiffness of the beam (Figure 6a) indicated a 1.5% reduction of the stiffness compared to initial condition. This was followed by a slight deviation with lower flexural stiffness to the maximum load, likely attributed to damage and possible softening of the structure. The corresponding evolution of the calculated strain energy (SE) and DDE with increasing beam deflection is shown in Figure 6b. A sudden load drop was observed at the maximum load, due to the composite rupture by fiber buckling in the first lamina under compression, as shown in Figure 6c.

Results show that a negligible amount of energy was dissipated prior to the beam deflection of 13.5 mm. This suggests that limited damage took place on the structure up to this critical load level. However, the rate of energy dissipation abruptly increased for larger composite beam deflection. Although an MD GFRP composite was considered under complex bending load, only the single damage mode for fiber failure occurred in the first lamina (0°) under compression, while other laminas remained elastic until the maximum load was experienced. The fiber damage initiation was predicted at 13.5 mm deflection. Based on the proposed method of specifying the yield point of the composite structure, yield began when the rate of the DDE increased to 0.914 N/(mm·s) at a 5% increase in the initial slope DDE evolution curve. Thus, this load (stress) and deflection level of (350 N, 13.5 mm) at which the rate of the DDE abruptly increased was identified as the yield point of the GFRP composite laminates. Deformation beyond the yield limit to failure was dominated by the softening of the structure. The total DDE of the GFRP composite corresponding to the maximum load at failure was 234 N/mm, which was 3.4% of the total SE of the structure at 6960 N/mm (Figure 6b). The flexural stiffness of the GFRP composite was reduced to 3% and 15.3% from the initial value, at the yield point and maximum load level, respectively.

### 5.2. Structural Response and Damage Evolution of CFRP Composite Beam under Three-Point Bending Load

**Figure 7:**
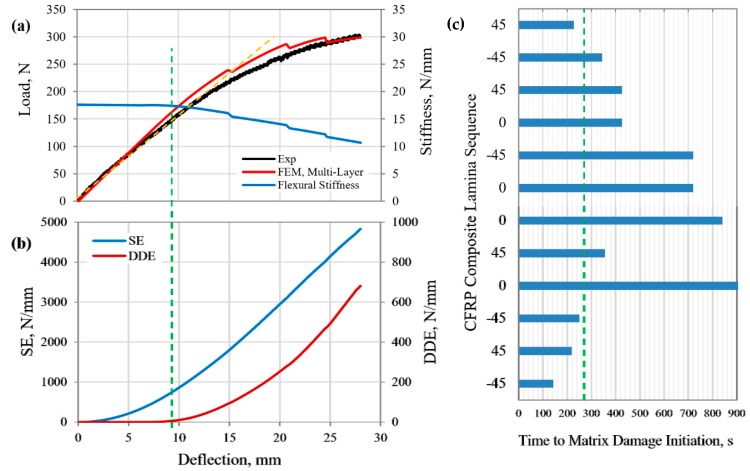
(**a**) Load–deflection response, structure stiffness curve, (**b**) strain and damage dissipation energies, and (**c**) time to matrix damage initiation in the laminas of CFRP composite beam under 3PB load.

The FE-calculated load–deflection response of the MD CFRP composite beam specimen (*Case 2*, Table 1) was compared with the measured curve, as shown in Figure 7a. A reasonably good prediction of the flexural response was claimed; thus, the validity of the FE simulation was ensured. Linear elastic response was observed up to a displacement of about 12.5 mm. A noticeable difference between the FE-predicted and measured deformation at larger deflection, with apparent minute load drops predicted along the load-displacement curve. Such a load drop was artificially induced by the relative slip between the CFRP composite laminate beam specimen and the support rollers under the assumed friction-free condition [45]. As described for the GFRP composite *(Case 1)* above, the observed reduction in the flexural modulus as reflected in the stiffness curve (Figure 7a) was due to the accumulated damage by the multiple modes of the failure of the composite constituents.

The corresponding evolution of the calculated DDE with increasing beam deflection, as shown in Figure 7b, exhibited identical characteristics to that of the GFRP composite *(Case 1).* Based on the proposed method of specifying the yield point of the composite structure, yield began when the rate of the DDE abruptly increased to 2.1 N/(mm·s) (i.e., 5% increase in the initial slope). Thus, the yield point was identified to correspond to the load and deflection levels of (150 N, 9 mm), when a sudden increase in the rate of the DDE was observed. It is worth mentioning that catastrophic fracture did not occur for this MD CFRP composite beam specimen at the maximum prescribed central displacement of 28 mm. However, extensive softening of the material by the various damage mechanisms was expected to have occurred. The FE model predicted various matrix cracking and crushing phenomena in different laminas with respect to the level of beam deflection, as shown in Figure 7c. The time to the onset of matrix cracking was predicted firstly in the bottom most lamina (−45°) of the composite beam when loaded up to 4.7 mm deflection, which represented the elastic deformation limit of the composite structure. The accumulated DDE at this displacement was 680 N/mm, which was 14% of the total strain energy of the composite structure (4825 N/mm, Figure 7b). The initial flexural stiffness of the CFRP composite beam of 17.6 N/mm was reduced to 17.44 and 10.66 N/mm (0.91% and 39.4% reduction) at the yield point and maximum load level, respectively.

### 5.3. Structural Response and Damage Evolution of CFRP Composite Beam under Four-Point Bending

**Figure 8:**
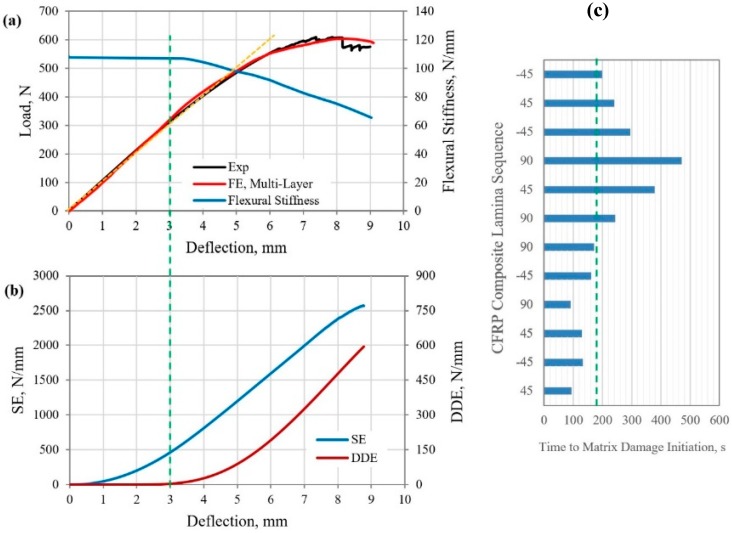
(**a**) Load–deflection response, structure stiffness curve, (**b**) strain and damage dissipation energies, and (**c**) time to matrix damage initiation in the laminas of CFRP composite beam under 4PB load.

The FE-calculated load–deflection response of the MD GFRP composite beam specimen (*Case 3*, Table 1) under 4PB was compared with the measured curve, as shown in Figure 8a. Again, a reasonably good comparison was demonstrated, and a valid FE simulation procedure of the test was claimed. A similar trend of the flexural deformation of the MD FRP composite beam specimens was observed in the three different specimens considered in this study. A larger applied total load of 600 N was recorded over a much shorter prescribed displacement of 8 mm, when compared with the 3PB *(Case 2*, Table 1) and different anti-symmetric lay-ups of the specimen. Linear elastic flexural response was measured up to the central deflection of about 4.6 mm, while the flexural stiffness curve (Figure 8a) was reduced by 7.1% from the initial condition. The corresponding evolution of SE and DDE in this antisymmetric CFRP composite beam specimen, with the applied displacement of the loading rollers, under the 4PB load, is shown in Figure 8b. Based on the proposed method of identifying the yield point of the composite, yield commenced when the rate of the DDE sharply rises to 11.1 N/(mm·s). The corresponding load and deflection level at yield were 313 N and 3 mm, respectively. It is worth noting that the load–deflection curve remained fairly linear beyond the yield point up to about 4.7 mm. Within this small deflection range, the rapid evolution of matrix damage in all of the laminas under tensile deformation (Figure 8c) only contributed to a fraction (about 10%) of the total DDE; thus, the effect on the softening of the material remained insignificant. A similar observation applied to the previous different FRP composite specimens and loading conditions, as described above. The accumulated DDE at the end of the prescribed displacement was 598.4 N/mm, which was 23% of the total SE of the composite beam (2567 N/mm, Figure 8b). The flexural stiffness of the CFRP composite was reduced by 1.1% and 31% of the initial value at the yield point and maximum load level (8-mm deflection), respectively.

### 5.4. Comparison of the Estimated Yield Limits Based on UD Hashin Criteria and Energy-Based Criterion

Three cases of FRP composite structure were tested under flexural loading condition, while the FE model was used to predict the material behavior and specify the structural yielding based on UD criteria, as well as the energy-based model proposed in this study. A summary of the results in terms of the maximum load and deflection capacities of the structures, yielding according to the UD criteria and energy-based criteria, and the percentage of the yield value to the maximum capacity (MC) of the structure is listed in Table 3.

Results indicated that, in the condition of the single failure mode (*Case 1*), the UD criteria (i.e., Hashin model) and energy-based criterion would suggest a similar range for yielding of the MD composite structure. However, MD composite structures mostly failed due to multiple damage phenomena (*Cases 2* and *3*), in which case, considering UD criteria would result in the assumption of structural yielding at only 10%–20% maximum capacity of the structure (displacement or load). While using the energy-based criterion, the yielding limit could safely (controlled condition in which the accumulation of all damage modes contributed to less than 5% of energy dissipation) increase to 30%–50% of the maximum capacity of the structure. The knowledge of the safe limit of structural yielding could be used for the optimum design of composite structures that are typically implemented under complex loading conditions.

## 6. Conclusions

A new criterion was proposed to identify the onset of yield in MD FRP composite laminate structures subjected to any type of loading condition. The new energy concept provides a significantly larger safe limit of yield for MD composite structures that normally fail due to multiple damage phenomena, in which the accumulation of energy dissipated due to all damage modes is less than 5% of the fracture energy required for the structural rupture. The criterion is based on the damage energy dissipated through the multiple softening processes of composite laminate materials under different mesoscale failure modes. The characteristic evolution of the DDE was calculated using a validated FE model with damage-based constitutive equations. The criterion was examined for antisymmetric MD GFRP and CFRP composite laminates under three- and four-point bending test configurations. The following can be concluded:

1. The yield point of the FRP composite laminate structures could be identified by a 5% increase in the initial slope of the DDE evolution curve with respect to the applied load parameters.
2. At the yield point, the extent of damage by the various modes depended on material, lay-ups, load, and test configurations.
3. The yield points of the MD GFRP and CFRP composite laminates (*cases 1*, *2*, and *3*) were identified to occur upon flexural loading when the rate of the DDE reached 0.914, 2.1, and 11.1 N/(mm·s), respectively. The corresponding deflections were 13.5, 9, and 3 mm, respectively.
4. The initial flexural stiffness of the MD GFRP and CFRP composite structures *(cases 1*, *2*, *and 3)* were measured at 28, 17.6, and 108.26 N/mm, reduced to 27.2, 17.44, and 107.1 N/mm at the yield point, indicating 3%, 0.91%, and 1.1% reductions in the stiffness of the beams, respectively. Therefore, an average 2% reduction in flexural stiffness could be suggested as a mean for the determination of the yield point in MD FRP composite structures under three- and four-point bending loads.
5. In general, the UD criteria resulted in the assumption of structural yielding at 10%–20% maximum capacity of the structure (displacement or load), whereas, using the energy-based criterion, the yield limit could be safely increased to 30%–50% of the maximum capacity of the structure.

## Figures and Tables

**Table 1:** Configuration of multidirectional (MD) fiber-reinforced polymer (FRP) composite specimens and loading type. ID—identifier; CF—carbon fiber; GF—glass fiber; PB—point bending.

Composite Panel (Case ID)	Laminate Sequences	Dimensions of the Beam Specimen (mm)					Loading Rate(mm/min)	Loading Type
		Length L	Width W	Laminate Thickness t	Lamina Thickness	Support Span Length L_SS_		
*GFRP* *(Case 1)*	[0/90/45/0/−45/90/45/0]	210	25	4	0.5	170	2	3PB
*CFRP* *(Case 2)*	[45/−45/45/0/−45/0 /0/45/0/−45/45/−45]	140	20	2.4	0.2	112	2	3PB
*CFRP* *(Case 3)*	[−45/45/−45/90/45/90/ 90/−45/90/45/−45/45]	70	20	2.4	0.2	60	1	4PB

**Table 2:** Elastic and damage properties of unidirectional (UD) GFRP and CFRP composite laminas.

Lamina Constants			Constitutive Damage Model Parameters of Lamina			
	GFRP	CFRP			GFRP	CFRP
E_11_, GPa	36.9	105.5	Longitudinal tensile strength, MPa	*X_T_*	820	1340
E_22_, GPa	10	7.2	Longitudinal compressive strength, MPa	*X_C_*	500	1192
E_33_, GPa	10	7.2	Transverse tensile strength, MPa	*Y_T_*	80.6	19.6
G_12_, GPa	3.3	3.4	Transverse compressive strength, MPa	*Y_C_*	322	92.3
G_13_, GPa	3.3	3.4	Longitudinal shear strength, MPa	*S_L_*	54.5	51
G_23_, GPa	3.6	2.52	Transverse shear strength, MPa	*S_T_*	161.2	23
ν_12_	0.32	0.34	Longitudinal tensile fracture energy, N/mm	*G_XT_*	32	48.4
ν_13_	0.32	0.34	Longitudinal compressive fracture energy, N/mm	*G_XC_*	20	60.3
ν_23_	0.44	0.378	Transverse tensile fracture energy	*G_YT_*	4.5	4.5
			Transverse compressive fracture energy, N/mm	*G_YC_*	4.5	8.5

**Table 3:** Results of the yield values (UD and energy-based criteria) of the MD composite structure under flexural loading condition.

Composite Panel (Case ID)	Yield Parameter	Maximum Capacity (MC)	Yield Point				Damage Type
			UD Hashin Criteria		Energy-based Criteria		
			Value	Percentage to MC	Value	Percentage to MC	
*GFRP* *(Case 1)*	Deflection, mm	23.4	13	55.5%	13.5	57.7%	Single mode Fiber failure in first lamina (0°)
	Load, N	554.7	337	60.7%	350	63.1%	
*CFRP* *(Case 2)*	Deflection, mm	28	**4.7**	**16.8%**	**9**	**32.1%**	Mixed-matrix cracking and crushing events in different laminas
	Load, N	301	**80.5**	**26.7%**	**150**	**49.8%**	
*CFRP* *(Case 3)*	Deflection, mm	8	**1.5**	**18.7%**	**3**	**37.5%**	
	Load, N	600	**161**	**26.8%**	**313**	**52.2%**	

## Abbreviations

$\left[\hat{\sigma }\right]$	Effective stress of lamina (MPa)
Y^T^	Transverse tensile strength (MPa)
S^L^	Longitudinal shear strength (MPa)
S^T^	Transverse shear strength (MPa)
Y^C^	Transverse compressive strength (MPa)
X^T^	Longitudinal tensile strength (MPa)
X^C^	Longitudinal compressive strength (MPa)
$d_{m}^{t}$	Matrix damage initiation variable in lamina under tensile load
$d_{m}^{c}$	Matrix damage initiation variable in lamina under compressive load
$d_{f}^{t}$	Fiber damage initiation variable in lamina under tensile load
$d_{f}^{c}$	Fiber damage initiation variable in lamina under compressive load
D	Damage operator in post-damage initiation process
$d_{f},{\;d}_{m}$, and $d_{s}$	Internal damage variable corresponding to lamina fiber, matrix, and shear damage modes
G_C_	Fracture energy (N/mm)
G^XT^	Longitudinal tensile fracture energy (N/mm)
G^XC^	Longitudinal compressive fracture energy (N/mm)
G^YT^	Transverse tensile fracture energy (N/mm)
G^YC^	Transverse compressive fracture energy (N/mm)
G_DDE_	Damage dissipation energy (N/mm)
*d_p_*	Damage evolution variable
$k_{eq}^{0}$	Equivalent elastic stiffness at the onset of damage (MPa)
$\delta_{eq}^{0}$	Equivalent displacement at the onset of damage (mm)
$\delta_{eq}^{f}$	Equivalent displacement at the separation of the material point (mm)
$\sigma_{ij}^{o},$$\tau_{ij}^{o},$ and $\varepsilon_{ij}^{o}$	Effective stresses (MPa) and strains at the onset of damage
$\delta_{\mathrm{eq}}^{0}$	Equivalent displacement at the state of damage initiation (mm)
$\delta_{\mathrm{eq}}^{f}$	Equivalent displacement at failure (mm)
$L^{c}$	Characteristic length of an element (mm)
$E_{U}$	Internal energy (N/mm)
$\sigma^{c}$	Stress (MPa)
${\dot{\varepsilon }}^{el},\;{\dot{\varepsilon }}^{pl},\;{\dot{\varepsilon }}^{cr}$	Time rate of elastic, plastic, and creep strains
$E_{S}$	Elastic strain energy (N/mm)
$\sigma^{u}$	Un-damaged stress (MPa)
$f^{u}$	Undamaged elastic energy function
$f^{c}$	Damage strain energy function
E_E_ and E_D_	Recoverable and irrecoverable energy (N/mm)
h and s	Interaction between the nodes-in-contact as separation and sliding motions (mm)
$N_{i}$	Interpolation function of the interface segments
$\rho_{n}$	Interface segment curvature
$D_{ij}$	Stiffness matrix of interface with linear coupled elastic behavior (MPa)
$F_{i}$	Force of interface node $i^{th}$ (N)
$u_{i}$	Motion of interface node $i^{th}$ (mm)
